# Prunetinoside Inhibits Lipopolysaccharide-Provoked Inflammatory Response via Suppressing NF-κB and Activating the JNK-Mediated Signaling Pathway in RAW264.7 Macrophage Cells

**DOI:** 10.3390/ijms23105442

**Published:** 2022-05-13

**Authors:** Abuyaseer Abusaliya, Pritam Bhagwan Bhosale, Hun Hwan Kim, Sang Eun Ha, Min Yeong Park, Se Hyo Jeong, Preethi Vetrivel, Joon-Suk Park, Gon Sup Kim

**Affiliations:** 1 Research Institute of Life Science, Department of Veterinary Medicine, Gyeongsang National University, Jinju 52828, Korea; yaseerbiotech21@gmail.com (A.A.); shelake.pritam@gmail.com (P.B.B.); shark159753@naver.com (H.H.K.); sangdis2@naver.com (S.E.H.); lilie17@daum.net (M.Y.P.); tpgy123@gmail.com (S.H.J.); 2 Department of Pharmacy, National University of Singapore, Singapore 117643, Singapore; preethiv@nus.edu.sg; 3 Preclinical Research Center, Daegu-Gyeonbuk Medical Innovation Foundation (DGMIF), Daegu 41061, Korea; jsp@kmedihub.re.kr

**Keywords:** prunetinoside, anti-inflammatory, NF-κB pathway, MAPK pathway

## Abstract

Inflammation is a multifaceted response of the immune system at the site of injury or infection caused by pathogens or stress via immune cells. Due to the adverse effects of chemical drugs, plant-based compounds are gaining interest in current research. Prunetinoside or prunetin-5-O-glucoside (PUG) is a plant-based active compound, which possesses anti-inflammatory effects on immune cells. In this study, we investigate the effect of PUG on mouse macrophage RAW264.7 cells with or without stimulation of lipopolysaccharide (LPS). Cytotoxicity results showed that PUG is non-cytotoxic to the cells and it reversed the cytotoxicity in LPS-stimulated cells. The levels of nitric oxide (NO) and interleukin-6 (IL-6) were determined using a NO detection kit and IL-6 ELISA kit, respectively, and showed a significant decrease in NO and IL-6 in PUG-treated cells. Western blot and qRT-PCR were performed for the expression of two important pro-inflammatory cytokines, COX2 and iNOS, and found that their expression was downregulated in a dose-dependent manner. Other pro-inflammatory cytokines, such as IL-1β, IL-6, and TNFα, had reduced mRNA expression after PUG treatment. Furthermore, a Western blot was performed to calculate the expression of NF-κB and MAPK pathway proteins. The results show that PUG administration dramatically reduced the phosphorylation of p-Iκbα, p-NF-κB 65, and p-JNK. Remarkably, after PUG treatment, p-P38 and p-ERK remain unchanged. Furthermore, docking studies revealed that PUG is covalently linked to NF-κB and suppresses inflammation. In conclusion, PUG exerted the anti-inflammatory mechanism by barring the NF-κB pathway and activating JNK. Thus, prunetinoside could be adopted as a therapeutic compound for inflammatory-related conditions.

## 1. Introduction

Inflammation is a natural process provoked by the invasion of microbes (virus, fungi, bacteria, and other pathogens), tissue damage, and even stress to maintain cell homeostasis. Inflammation is accompanied by pathological reactions and multifaceted responses taking place in the infected tissues, injured cells, or toxin-affected microenvironments [1] and ends in severe tissue damage, with the association of pro-inflammatory cytokines production in case of chronic inflammation [2]. Commonly, inflammation reactions happen in an independent manner, increasing and reducing the synthesis of the anti-inflammatory and pro-inflammatory mediators, respectively [3]. However, in the case of a chronic inflammatory reaction, the process will be reversed, where pro-inflammatory mediators will be increased while anti-inflammatory mediators will get decreased [4,5]. The inflammation arises from the activation of the immune cells, including macrophages, neutrophils, and lymphocytes; among these, macrophages play a vital role in the inflammatory response [6].

Macrophages are immune cells and an active contributor to innate immunity by scavenging potent pathogens. During the inflammatory process, macrophages are activated and eliminate the harmful allergens from the body [7]. Stimulation of macrophages results in the synthesis of inflammatory mediators [8]. In general, the synthesis of pro-inflammatory mediators, such as inducible nitric-oxide synthase (iNOS), cyclooxygenase 2 (COX2), and nitric-oxide (NO) [9], as well as pro-inflammatory cytokines, such as tumor necrosis factor (TNF-) and interleukin-6 (IL-6), as a reaction to inflammation at injured sites, is linked to the inflammation response. NO release is closely connected with iNOS during the inflammation process [10]. Furthermore, IL-6 has a significant function in macrophage survival and is also involved in the etiology of inflammatory and malignant diseases [11]. The expression of iNOS during the inflammatory process is closely connected with nuclear factor-κB (NF-κB).

NF-κB is the foremost transcription factor that regulates inflammatory gene expression of iNOS, IL-8, IL-6, and TNF-α [12,13]. This pathway is activated by stress or signals from pathogens and activates its family proteins, p65 (Rel-A), NF-κB, Rel-B, and c-Rel. Studies have shown that the NF-κB family of proteins has a starring role in the host defense mechanism against stress or pathogens [14]. Mitogen-activated protein kinases (MAPKs) will be activated by stresses and are highly associated with inflammation reactions. ERK, p38, and JNK are the three kinases that convert extracellular signals into intracellular pathways and lie in the protein kinase cascades [15,16]. In the regulatory mechanism of COX2 expression and other cytokines, MAPKs are the vital element [17], and previous studies show that MAPK is a key factor involved in the inflammatory response [18,19,20]. The c-Jun N-terminal kinase (JNK) signaling cascade of MAPK is involved in the production of cytokines and the upregulation of COX2 in chronic inflammatory diseases, and JNK activation is supplementary to the production of NO and IL-6 [21]. IL-6, NF-κB, and JNK signaling has risen to prominence as a potential therapeutic target in inflammatory diseases.

**Figure 1:**
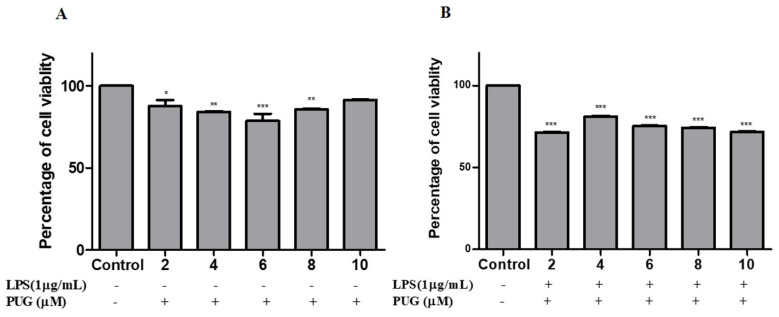
Cytotoxicity effect of PUG on RAW 264.7 cells. Pretreatment with or without LPS (1 μg/mL) on cells for 1 h at 37 °C. Then, cells were treated with PUG (0, 2, 4, 6, 8, and 10 μM) for 24 h at 37 °C. (**A**) Cytotoxicity effect of PUG on non-LPS-induced RAW264.7 cells. (**B**) Cytotoxicity effect of PUG on LPS-induced cell viability in RAW264.7 cells. The data are presented as the mean ± standard error of the mean (SEM). * *p* < 0.05, ** *p* < 0.01, *** *p* < 0.001.

Flavonoids are bioactive compounds from plants that possess various activities, including anti-inflammatory and anti-cancer. Prunetinoside (prunetin-5-O-glucoside, or PUG) (Figure 1) is a flavonoid (flavonol) from *Prunus* spp. and *Betula* spp. A *Prunus yedoensis* bark crude extract study revealed that it has anti-inflammatory properties in adipose tissue of obese induced mice [22]. PUG has antiproliferative activity and arrests at the G2 phase of the cell cycle in the AGS gastric cancer cell line [23]. The anti-inflammatory activity of PUG in RAW264.7 cells and its mechanism of action are still unknown. Thus, we aim to explore the anti-inflammatory effect of PUG on RAW 264.7 macrophage cells.

## 2. Results

### 2.1. Cell Cytotoxicity Effect of PUG on Activated Macrophage

After pretreatment with and without LPS (1 μg/mL) for 1 h, cells were treated with gradual doses of PUG (0, 2, 4, 6, 8, and 10 μM) for 24 h and the effect was assessed using an MTT assay. The result, shown in Figure 1, suggests that even up to 8 μM concentration PUG was non-toxic to the non-LPS treated cells. Hence, doses of 4 and 6 μM concentrations were used for subsequent experiments.

### 2.2. Morphological Hallmarks Associated with PUG in Macrophage Cells

**Figure 2:**
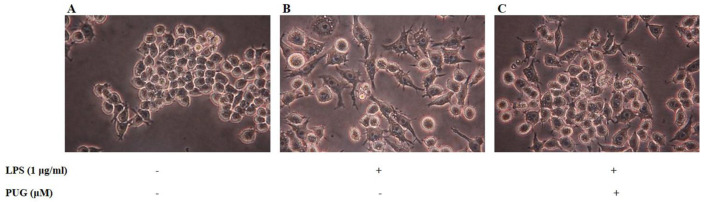
Morphological changes induced by PUG in RAW264.7 cells: Cells were grown in DMEM media at 37 °C in a humidified environment with 5% CO_2_. After attaining the desired growth, the cells were seeded at 5 × 10^4^ cells/plate. Then, LPS (1 μg/mL) treatment for 1 h, followed by PUG (6 μM) treatment for 24 h were given. After treatment, plates were observed under an inverted microscope. (**A**) Control cells. (**B**) LPS-stimulated cells. (**C**) LPS + PUG (6 μM) co-treated cells.

To picturize the morphological changes, cells were treated with LPS (1 μg/mL) for 1 h and co-treated the cells with LPS + PUG with different concentrations and observed under a light microscope. When treated with LPS alone, the cells tended to swell, elongate, and increase their cell volume compared to control cells (Figure 2A), which indicates the cells have been stimulated and inflammation has occurred (Figure 2B). On the other hand, in cells in the co-treated group with LPS + PUG, swelling and cell size were comparatively reduced, which indicates that PUG reversed the process and the cells recovered from the inflammation (Figure 2C).

### 2.3. Effect of PUG on Nitric Oxide (NO) Release on RAW264.7 Macrophage Cells

**Figure 3:**
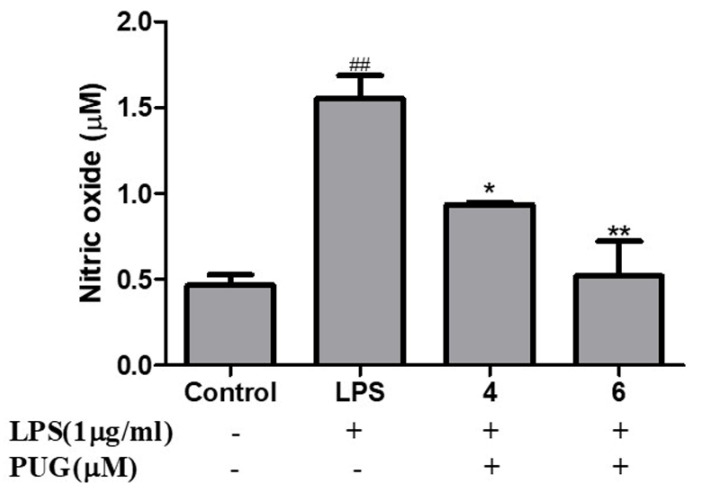
Effect of PUG on nitric oxide (NO) release on RAW264.7 macrophage cells. RAW264.7 cells were pretreated without and with LPS (1 μg/mL) for 1 h at 37 °C. Then the cells were treated with PUG (4, and 6 μM) for 24 h at 37 °C. After treatment, the medium was collected and used for the assay. A NO detection kit was used to determine the NO concentration in the samples. The samples were measured at 520 nm. The absorbance values of the standards and samples were used to plot the graph. The statistical significance was ## *p* < 0.01 vs. untreated group; and * *p* < 0.05, ** *p* < 0.01 vs. LPS-alone treated group.

The effect of PUG on nitric oxide content in the cell supernatant was estimated with Griess Reagent. Pre-treatment with and without LPS (1 μg/mL) was for 1 h at 37 °C, followed by treatment for 24 h at 37 °C with a progressive concentration of PUG. As shown in Figure 3, when related to the control, the LPS treatment led to an observable increase in NO production and it was significantly suppressed by the PUG treatment in a dose-dependent manner for both doses.

### 2.4. Effect of PUG on the Pro-Inflammatory Cytokine Interleukin-6 (IL-6) on Activated RAW264.7 Macrophage Cells

**Figure 4:**
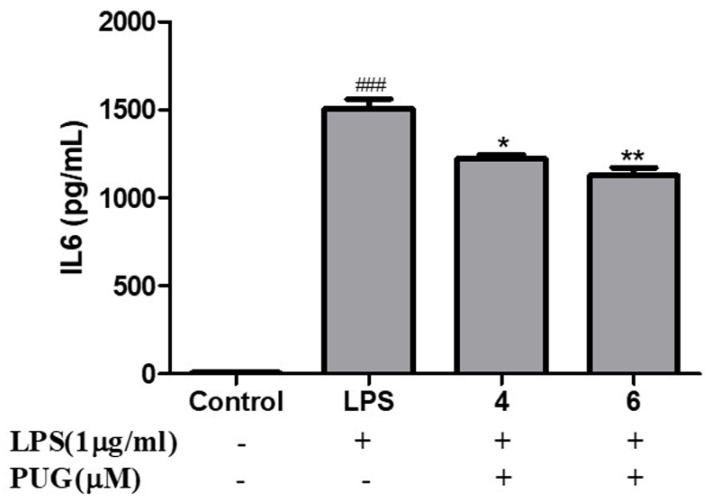
Effect of PUG on the pro-inflammatory cytokine interleukin-6 (IL-6) on activated RAW264.7 macrophage cells. RAW264.7 cells were pretreated without or with LPS (1 μg/mL) for 1 h at 37 °C. Then the cells were treated with PUG (4, and 6μM) for 24 h at 37 °C. The treated cell culture media were collected and used for the IL-6 determination. The wells were read, and absorbance values were used for the graph plot. The standard deviation and error were calculated from the replicates of the samples and the Four Parameter Logistic curve (4PL) method was used to determine the IL-6 concentration. The statistical significance was ### *p* < 0.001 vs. untreated group; and * *p* < 0.05, ** *p* < 0.01 vs. LPS-alone treated group.

To determine the effect of PUG on IL-6 of LPS-activated RAW264.7 cells, the cells were pretreated with LPS (1 μg/mL) for 1 h and co-treated with LPS + PUG (4 and 6 μM), and the cell supernatant was collected and IL-6 was measured using an IL-6 ELISA Kit. The results show that notably there is an increased level of IL-6 in the LPS treatment compared to the control group, but IL-6 release was dose-dependently inhibited in the PUG-treated cells (Figure 4). It confirms that PUG has anti-inflammatory effects by inhibiting the pro-inflammatory cytokine IL-6.

### 2.5. PUG Decreases the mRNA Expression of Pro-Inflammatory Cytokines in Activated RAW264.7 Macrophage Cells

**Figure 5:**
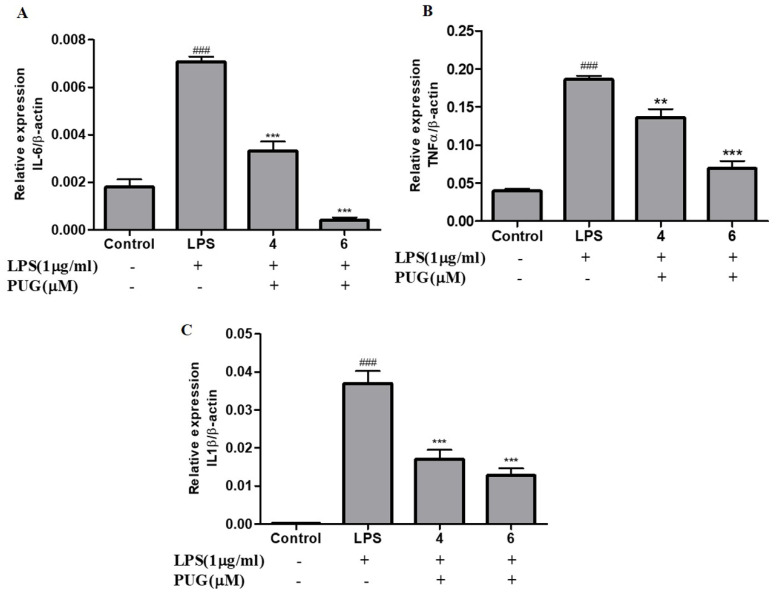
PUG decreases the protein expression of pro-inflammatory cytokines in activated RAW264.7 cells: The cells were treated with LPS (1 μg/mL) and PUG (4, and 6 μM) for 1 h and 24 h, respectively. After treatment the RNA was isolated from all the groups and converted into cDNA. Then the converted cDNA was used for qRT-PCR with the kit components. The graph was plotted using C_q_ values by the 2^−ΔΔCq^ method. The relative expression of each gene was calculated by normalizing with the β-Actin gene. (**A**) Relative expression of IL-6. (**B**) Relative expression of TNFα. (**C**) Relative expression of IL-1β. The statistical significance was ### *p* < 0.001 vs. untreated group; and ** *p* < 0.01, *** *p* < 0.001 vs. LPS-alone treated group.

The level of mRNA expression of pro-inflammatory cytokines, including IL-1β, IL-6, and TNFα, in RAW264.7 cells were analyzed using qRT-PCR. As shown in Figure 5, the expression level on the LPS-stimulated cells significantly increased compared to the control cells. The expression levels of all the cytokines, IL-6 (Figure 5A), TNFα (Figure 5B), and IL-1β (Figure 5C), were commendably decreased in the PUG-treated group in a dose-dependent manner. These results confirm that PUG inhibits the pro-inflammatory cytokines at the transcription level.

### 2.6. PUG Decreases the Expression of Pro-Inflammatory Cytokines iNOS and COX2 in Activated RAW264.7 Cells

**Figure 6:**
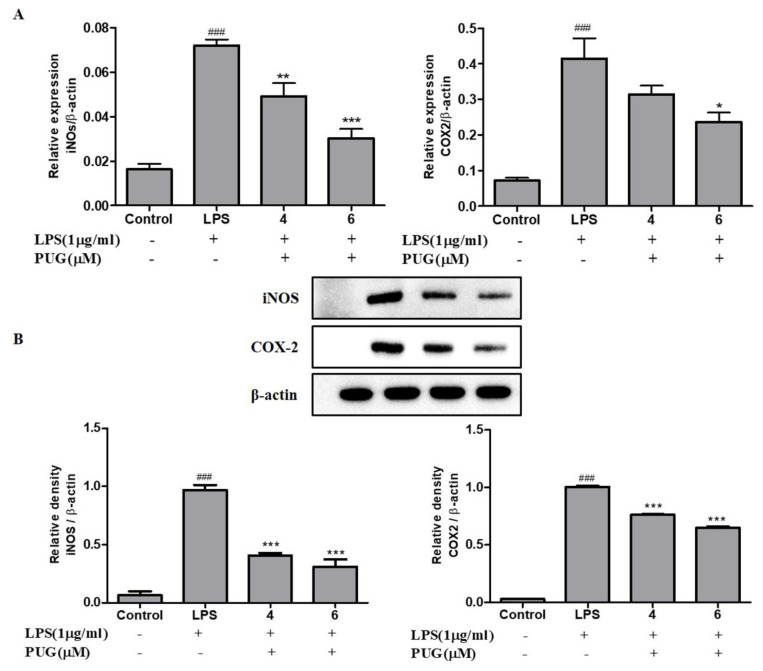
PUG decreases the mRNA and protein expression of pro-inflammatory cytokines COX2 and iNOS in activated RAW264.7 macrophage cells: (**A**) mRNA expression of COX2 and iNOS; (**B**) Western blot analysis of COX2 and iNOS. For mRNA expression, the RNA was isolated from the cells after treatment and converted into cDNA. The converted cDNA was used for the quantitative expression. The Cq values were used for plotting the graph by the 2^−ΔΔCq^ method. The relative expression for both genes was calculated by normalization with the β-actin gene. For protein expression, the protein was harvested from the cells and Western blotting was performed. The expression levels were calculated by the densitometry with the SEM of three independent values. The statistical significance was ### *p* < 0.001 vs. untreated group; and * *p* < 0.05, ** *p* < 0.01, *** *p* < 0.001 vs. LPS-alone treated group.

The pro-inflammatory cytokine’s mRNA expression of COX2 and iNOS was analyzed using qRT-PCR; similarly, the protein expression levels were evaluated using Western blotting. The expression of both iNOS and COX2 were significantly increased in LPS-stimulated cells compared to the control. Conversely, the stimulated iNOS and COX2 expression were significantly downregulated upon PUG treatment in both mRNA (Figure 6A) and protein (Figure 6B) in a dose-dependent manner. This suggests that PUG downregulates the expression levels of iNOS and COX2 at both the transcription and translation levels.

### 2.7. The Effect of PUG on the NF-κB Pathway

**Figure 7:**
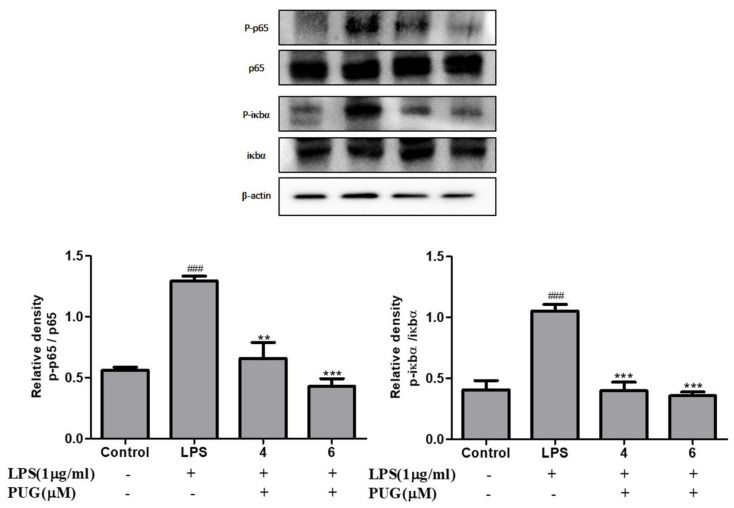
PUG inhibits the NF-κB pathway in LPS-stimulated RAW264.7 cells: Western blot analysis of the NF-κB pathway proteins. After the treatment with LPS and PUG, the protein was isolated from the cells and Western blotting was performed. The expression levels were calculated by the densitometry with the SEM of three independent values. The relative density was calculated with the total form and phospho forms of the protein. The statistical significance was ### *p* < 0.001 vs. untreated group; and ** *p* < 0.01, *** *p* < 0.001 vs. LPS-alone treated group.

The effect of PUG on NF-κB in LPS-stimulated RAW264.7 cells was examined by Western blotting. After pre-treatment with LPS for 1 h, the phosphorylation of IκBα and NF-κB 65 were significantly increased compared to the non-LPS treatment. In addition, PUG treatment reversed the expression level of p-Iκbα and p-NF-κB 65 significantly in a dose-dependent manner (Figure 7). The expression levels of p-Iκbα and p-NF-κB 65 were normalized with Iκbα and NF-κB 65, respectively. Thus, the result proves PUG possesses an anti-inflammatory property by inhibiting the NF-κB pathway in LPS-induced RAW264.7 macrophage cells.

### 2.8. Effect of PUG on Activating MAPKs in LPS Stimulated RAW264.7 Cells

**Figure 8:**
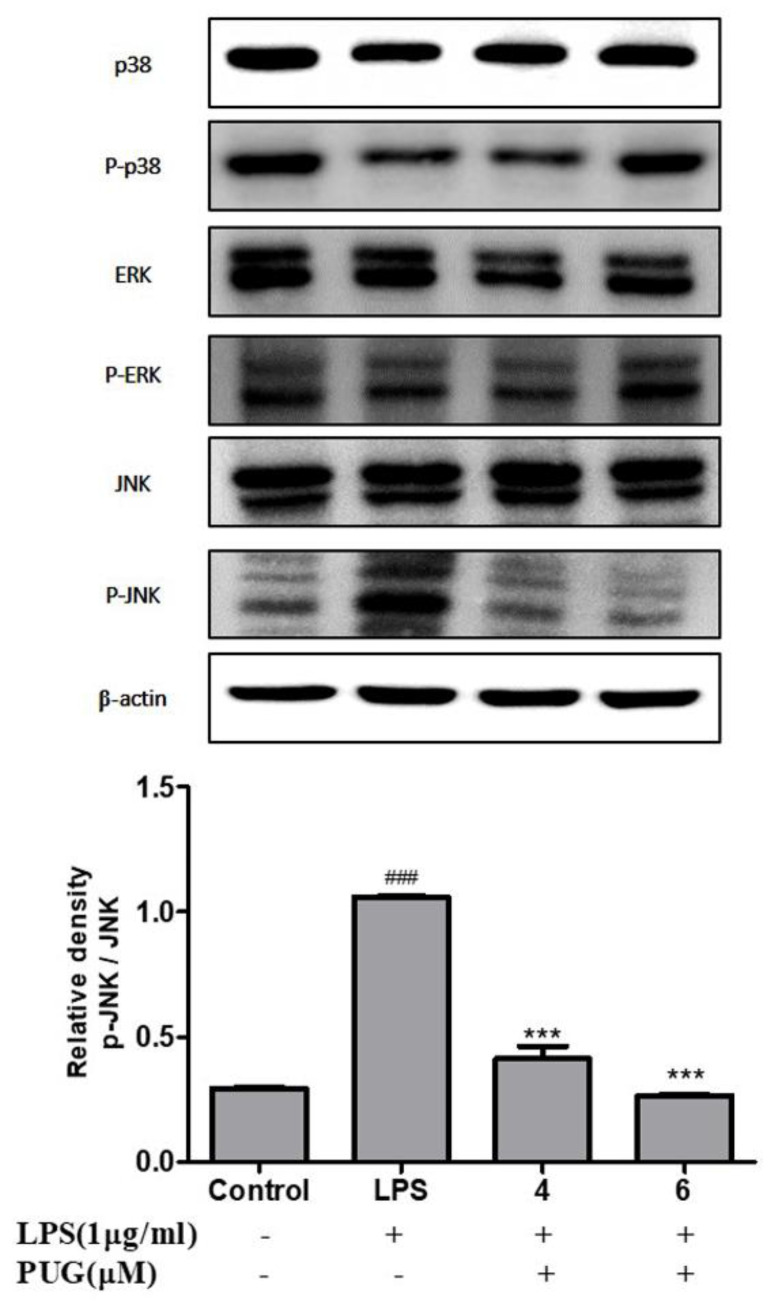
Effect of PUG on activating MAPKs in LPS-stimulated RAW264.7 cells: RAW264.7 cells were pretreated without or with LPS (1 μg/mL) for 1 h at 37 °C. Then the cells were treated with PUG (4, and 6 μM) for 24 h at 37 °C. After treatment, the protein was harvested from the cells and a Western blot was performed. The values were calculated by densitometry and the relative density was calculated with the total form of the protein. Three independent values were used for the calculation. The statistical significance was ### *p* < 0.001 vs. untreated group; and *** *p* < 0.001 vs. LPS-alone treated group.

Next, we examined the effect of PUG on the activation of MAP kinase protein expression in LPS-stimulated RAW264.7 cells. As shown in Figure 8, in the LPS-treated cells, the phosphorylation of JNK was significantly increased compared to the control. However, increased p-JNK was significantly reversed by dephosphorylation upon the PUG treatment in a dose-dependent manner. Interestingly, co-treatment of LPS-PUG did not affect the p-p38 and p-ERK, which remained the same. Taken together, this result indicates that the anti-inflammatory response of PUG on LPS stimulated cells has been mediated through the JNK signaling pathway.

### 2.9. Molecular Docking Analysis of PUG with NF-κB

**Figure 9:**
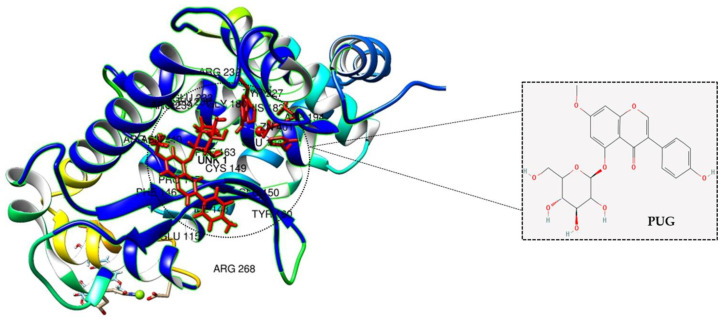
Molecular docking analysis of PUG with NF-κB: The ligand PUG (red) competently binds to the NF-κB protein complex with its interrelated amino acids CYS, PHE, PRO, ARG, TYR, GLU, and GLY.

For a better understanding of the inhibitory action of PUG on NF-κB, the PUG ligand was docked into the active site of the structure NF-κB protein. Upon docking, the chimera model with the lowest binding energy level was selected. The result indicated that PUG binds to the NF-κB protein with the binding free energy of −7.8 kcal/mol, and PUG relied mostly on hydrogen bonds for the interaction with NF-κB (Figure 9). ARG, GLU, CYS, PHE, PRO, GLY, and TYR are the amino acids that interact with PUG-NF-κB via hydrogen bonds and Van der Walls forces. This strongly suggests that PUG binds to the NF-κB protein complex and prevents it from transmitting signals.

## 3. Discussion

Plant-based or plant-derived drugs are appealing to drug discovery and industry due to their non-toxicity and lack of side effects [24]. Plants are a rich source of bioactive compounds, such as flavonoids, alkaloids, and phenols, which possess effective pharmacological effects such as being anti-diabetic, anti-cancer, anti-inflammation, antioxidant, anti-bacterial, and cardio-protective [25]. Several studies provide evidence that plant-derived glycosidic compounds have numerous biological activities, especially in inflammation and cancer [26]. For the therapeutic approach, an ideal compound should have the ability to downregulate the pro-inflammatory cytokines, block the NF-κB, and activate any signaling pathway. In this regard, prunetinoside (prunetin-5-O-glucoside) is a glycosidic flavonoid that is proven to have an anti-cancer effect. However, the anti-inflammatory effects of PUG are still unknown.

Inflammation is an innate immunity of the body and immune cells engaged in a defense mechanism to heal and protect the body from harmful infection. During this process, the immune cells release some chemical substances termed inflammatory mediators. Macrophages are the important cells tangled in an inflammatory response. Our MTT results show that the PUG is non-toxic to the macrophages at ≤10 μM and after LPS-PUG co-treatment, the cells were viable even at higher concentrations. Hence, 4 and 6 μM concentrations were chosen for the further experiments. Oxidative stress is an accumulation of reactive oxygen species (ROS) and will lead to the development of inflammatory diseases and NO is responsible for swelling and redness at the site of inflammation [27]. Thus, inhibiting nitric oxide (NO) production is one of the strategies to develop anti-inflammatory responses. In our study, we have determined that LPS stimulation has raised the NO levels and PUG co-treatment has inhibited the NO production in a dose-dependent manner. Interleukin-6 (IL-6) is one of the cytokinesis that releases prostaglandin E2 and IL-6 excites energy mobilization, which raises the body temperature. IL-6 has a key role in the production of inflammatory cytokines and NF-κB. In our study, an ELISA assay was performed and showed significant inhibition of the IL-6 synthesis in PUG-treated RAW264.7 cells in a dose-dependent manner.

Pro-inflammatory cytokines are the immune markers of any immune response, including IL-1β, IL-6, TNFα, COX2, and iNOS. Pro-inflammatory cytokines are involved in cell-mediated immune responses and play a vital role in modulating the immune system [28]. During inflammation, these cytokines will get activated at the inflammatory site. Our results of qRT-PCR demonstrated that all the pro-inflammatory cytokines were downregulated at the mRNA level, which indicates PUG inhibits pro-inflammatory cytokines and exerts an anti-inflammation response. Similarly, the protein expression of the two important pro-inflammatory cytokines, COX2 and iNOS, were also downregulated in a dose-dependent manner, lending support to the mRNA findings.

Previous works of literature show strong evidence of medicinal plant compounds with potential NF-κB-modulating activity. NF-κB is a transcriptional factor that can regulate genes that are mostly involved in immune response and progression of a cell [29,30]. The ubiquitination, phosphorylation, and proteolytic cleavage of NF-κB will trigger the IκBα and p65 proteins of the NF-κB signaling pathway and these two proteins will activate cytokines such as IL-6, IL-1β, and TNFα. In most of the reported inflammatory studies, the anti-inflammatory compound tended to block the NF-κB signaling pathway and exerted its mechanism [10,31]. Likewise, our protein expression results showed that after LPS treatment, the phosphorylated IκBα and p65 were elevated, and upon PUG co-treatment, phosphorylation of IκBα and p65 were downregulated, while the total forms of IκBα and p65 remains the same. This result strongly indicates PUG is an anti-inflammatory compound that can block the NF-κB signaling pathway.

When there is a crosstalk between NF-κB and MAPKs, MAPK is closely associated, as well as activating the NF-κB pathway and the primary role of MAPKs is to control cytokine expression [32]. Rationally, PUG’s pre-from, called prunetin, activates only the p-JNK and p-38 in the stimulated RAW264.7 cells [33]. Therefore, we examined the effect of PUG on MAPK proteins. Interestingly the results showed that PUG treatment downregulated the JNK dephosphorylation upon the concentrations of PUG. On the other hand, ERK or p38 remained the same, which is similar to the results of B. Cai et al. [21]. This corroborates that PUG blocks NF-κB signaling and activates JNK-MAPK to mediate the anti-inflammatory mechanism. Molecular docking analysis serves as a promising tool in drug discovery and structural molecular biology where the interaction between a ligand and a protein can be analyzed at the atomic level [34,35]. Thus, as a supportive piece of evidence on the suppression of NF-κB by PUG, the interaction between the molecular structure of NF-κB and PUG was analyzed by docking and the results showed that PUG had an efficient binding affinity with minimal energy to the NF-κB protein complex.

## 4. Materials and Methods

### 4.1. Cell Line Maintenance and Compound

Mouse macrophage RAW264.7 cells was purchased from ATCC and maintained in Dulbecco’s Modified Eagle Medium (DMEM) with 10% activated fetal bovine serum (FBS) (Gibco; Thermo Fisher Scientific, Seoul, Korea), 100 g/mL streptomycin, and 100 U/mL penicillin (Gibco; Thermo Fisher Scientific, Seoul, Korea). The cell development was kept at 37 °C in a humidified environment with 5% CO_2_. Prunetinoside was purchased commercially and prepared in a stock (20 M/mL) with DMSO. Then the desired concentration for the cell line treatments were diluted with DMSO.

### 4.2. Cytotoxicity Test

RAW 264.7 cells were seeded at a density of 1 × 10^4^ cells/well in a 96-well plate. The cells were pre-treated with and without LPS (1 μg/mL; Sigma-Aldrich, Seoul, Korea) for 1 h at 37 °C, and then treated for 24 h at 37 °C with a progressive concentration of PUG (2, 4, 6, 8, and 10 μM). After the treatment, to all wells were added MTT solution (10 μL; 5 mg/mL) and kept for roughly 4 h. Then, the well components were fully removed after incubation, and the insoluble formazan crystals were dissolved by adding DMSO and read using a Microplate photometer at 590 nm wavelength (Thermo Scientific).

### 4.3. Nitric Oxide (NO) Assay

For the NO assay, cells were seeded at a cell density of 1 × 10^4^ and then underwent a 1 h at 37 °C pre-treatment with and without LPS (1 μg/mL; Sigma-Aldrich), followed by 24 h of treatment with 4 and 6 μM of PUG at 37 °C. After the treatment period, 100 μL of supernatant from each well was used and analyzed with a NO Plus detection kit (iNtRON Biotechnology; Cat. No. 21023). Following the manufacturer’s instructions, 50 μL of N1 and N2 buffer was added. A PowerWave HT microplate spectrophotometer was used to read the reaction mixture at 520 nm (BioTek Instruments, Inc., Winooski, VT, USA). The curve was plotted with standard sodium nitrite and the NO concentration in each group was calculated using the standard.

### 4.4. Enzyme-Linked Immunoassay (ELISA)

RAW 264.7 cells were seeded at a cell density of 5 × 10^4^ in a 24-well plate. Then subjected to pre-treatment for 1 h at 37 °C of with and without LPS (1 μg/mL; Sigma-Aldrich) and PUG treatment with 4 and 6 μM for 24 h at 37 °C. The supernatant from the wells was collected and used for the determination of IL-6. The level of IL-6 was quantified by using a mouse IL-6 ELISA kit (Cat. No. ADI-900-045; Enzo Life Sciences, Inc., New York, NY, USA) rendering to the maker’s instructions. Four Parameter Logistic curve (4PL) method was used to plot the graph.

### 4.5. Real-Time Quantitative Reverse Transcription PCR (qRT-PCR)

For mRNA expression, RAW264.7 cells were grown at a density of 5 × 10^4^ cells/well in a 6-well plate and then subjected to pre-treatment for 1 h at 37 °C of with and without LPS (1 μg/mL; Sigma-Aldrich) and PUG treatment with 4 and 6 μM for 24 h at 37 °C. The total RNA was extracted by Trizol^®^ (Thermo Fisher Scientific, Inc.) reagent after treatment. The iScriptTM cDNA synthesis kit (BioRad Laboratories, Inc., Hercules, CA, USA) was used to convert isolated total RNA (1 μg) to cDNA rendering to the company’s instructions. The converted cDNA was then used to perform qPCR using the AccuPower^®^ 2X GreenstarTM qPCR Master mix (Bioneer Corporation), with the following reaction conditions: Pre-denaturation for 2 min at 95 °C, followed by 40 cycles of denaturation and annealing/extension at 95 °C for 5 s, at 58 °C for 30 s, respectively, in the CFX384 Real-Time PCR Detection System (BioRad Laboratories, Inc.). The qRT-PCR primers used for this study are given in Table 1.

### 4.6. Western Blotting

To determine the protein expression, RAW264.7 cells were seeded at a cell density of 5 × 10^4^ cells/plate in a 60π plate, and then subjected to pre-treatment for 1 h at 37 °C with and without LPS (1 μg/mL; Sigma-Aldrich) and PUG treatment with 4 and 6 μM for 24 h. After this, RIPA buffer (iNtRON Biotechnology, Incheon, Korea) was added to the cells to lyse and harvest the protein. Then the concentration of protein was estimated by the Pierce™ BCA protein assay kit (Thermo Scientific). To separate the proteins, an equal concentration of protein (10 μg) was loaded in SDS-PAGE. Then the gel was transferred to PVDF membranes via a Semi-Dry Transfer unit (Atto Corporation, Tokyo, Japan). The transferred membrane was then blocked for 1–2 h at room temperature with 5% Bovine Serum Albumin (BSA) and followed by incubation at 4 °C overnight with diluted (1:1000) primary antibodies. The membranes were washed with 1X TBST solution and probed with the horseradish peroxidase-conjugated secondary antibody (1:5000) anti-mouse (cell signaling; cat. No. A90-116P) for β-actin and anti-rabbit (cell signaling; cat. No. A120-101P) to the rest of the proteins at room temperature for 2 h and washed again. Finally, the blots were detected under detection system (Bio-Rad Laboratory), after developing the ECL solution. Protein densitometry was evaluated using the ImageJ software program.

### 4.7. Molecular Docking

For docking analysis, the protein structure of NF-κB was obtained from Protein Data Bank (PDB) (https://www.rcsb.org/) (accessed on 23 February 2022) (PDB ID—4Q3J) and the 3D structure of the compound prunetinoside was obtained from PubChem (https://pubchem.ncbi.nlm.nih.gov/) (accessed on 23 February 2022) (Compound CID: 44257332). The target protein and ligand were docked using USCF Chimera software (https://www.cgl.ucsf.edu/chimera/) (accessed on 23 February 2022)) with default parameters and obtained all the possible conformations. The estimated free energy of the binding and total intermolecular energy were used to evaluate the results.

### 4.8. Statistical Analysis

For mRNA expression, relative expression was determined by the 2^−ΔΔCq^ method [36]. All data were represented as the mean ± SD (Stranded Deviation). The statistical significance was # *p* < 0.05, ## *p* < 0.01, ### *p* < 0.001 vs. untreated group; and * *p* < 0.05, ** *p* < 0.01, *** *p* < 0.001 vs. LPS-alone treated group and determined using the Newman–Keuls Multiple Comparison Test.

## 5. Conclusions

**Figure 10:**
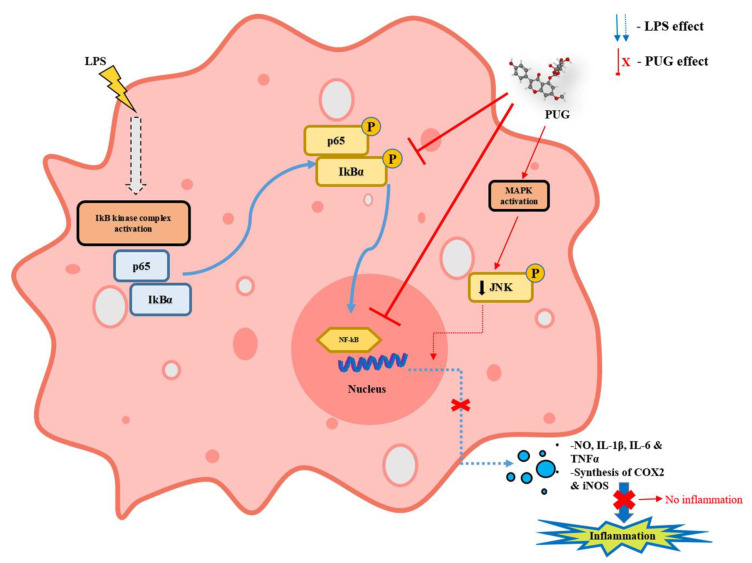
Schematic illustration of the anti-inflammatory action of PUG in macrophage RAW264.7 cells.

In conclusion, PUG a flavonoid has been found to inhibit inflammation in macrophages by significantly lowering the pro-inflammatory mediators such as iNOS, COX2, NO, and IL-6. Further, the mechanism of anti-inflammation mediated by PUG has been identified to be the inhibition of NF-κB signaling followed by successive downregulation of the pJNK/JNK-pathway, as illustrated in Figure 10. This study suggests that PUG possesses anti-inflammatory action in RAW264.7 macrophage cells and, as an alternative to chemotherapeutics, it could be utilized as an anti-inflammatory medication to treat inflammatory conditions.

## Figures and Tables

**Table 1:** Details of qRT-PCR primer used in this study.

Gene	Primer	Sequence (5′ to 3′)
iNOS	F	TCCTACACCACACCAAAC
	R	CTCCAATCTCTGCCTATC
COX2	F	CCTCTGCGATGCTCTTCC
	R	TCACACTTATACTGGTCAAATCC
IL-6	F	GAGGATACCACTCCCAACAGACC
	R	AAGTGCATCATCGTTGTTCATACA
IL-1β	F	TGCAGAGTTCCCCAACTGGTACATC
	R	GTGCTGCCTAATGTCCCCTTGAATC
TNFα	F	TGGAGTCATTGCTCTGTGAAGGGA
	R	AGTCCTTGATGGTGGTGCATGAGA
β-Actin	F	TACTGCCCTGGCTCCTAGCA
	R	TGGACAGTGAGGCCAGGATAG

## Data Availability

The data used to support the findings of this study are available upon request from the corresponding author.
